# Recalibrated Tree of Leaf Beetles (Chrysomelidae) Indicates Independent Diversification of Angiosperms and Their Insect Herbivores

**DOI:** 10.1371/journal.pone.0000360

**Published:** 2007-04-11

**Authors:** Jesús Gómez-Zurita, Toby Hunt, Fatos Kopliku, Alfried P. Vogler

**Affiliations:** 1 Department of Entomology, Natural History Museum, London, United Kindgom; 2 Division of Biology, Imperial College London, Ascot, United Kindgom; 3 Zoologische Staatssammlung München, Munich, Germany; Lund University, Sweden

## Abstract

**Background:**

The great diversity of the “Phytophaga” (weevils, longhorn beetles and leaf beetles) has been attributed to their co-radiation with the angiosperms based on matching age estimates for both groups, but phylogenetic information and molecular clock calibrations remain insufficient for this conclusion.

**Methodology:**

A phylogenetic analysis of the leaf beetles (Chrysomelidae) was conducted based on three partial ribosomal gene markers (mitochondrial *rrnL*, nuclear small and large subunit rRNA) including over 3000 bp for 167 taxa representing most major chrysomelid lineages and outgroups. Molecular clock calibrations and confidence intervals were based on paleontological data from the oldest (K-T boundary) leaf beetle fossil, ancient feeding traces ascribed to hispoid Cassidinae, and the vicariant split of Nearctic and Palearctic members of the Timarchini.

**Principal Findings:**

The origin of the Chrysomelidae was dated to 73–79 Mya (confidence interval 63–86 Mya), and most subfamilies were post-Cretaceous, consistent with the ages of all confirmed body fossils. Two major monocot feeding chrysomelid lineages formed widely separated clades, demonstrating independent colonization of this ancient (early Cretaceous) angiosperm lineage.

**Conclusions:**

Previous calibrations proposing a much older origin of Chrysomelidae were not supported. Therefore, chrysomelid beetles likely radiated long after the origin of their host lineages and their diversification was driven by repeated radiaton on a pre-existing diverse resource, rather than ancient host associations.

## Introduction

The Coleoptera (beetles) represent one of the most diversified lineages on Earth, with about 350,000 species described and total numbers probably an order of magnitude higher [1], [2]. Among beetles, the “Phytophaga” constitutes the largest radiation, representing roughly 40% of all known species [3]. This megadiverse lineage includes Curculionoidea (weevils) and Chrysomeloidea. The latter combines the Cerambycidae (longhorn beetles), usually with wood-boring larvae, and the Chrysomelidae *sensu lato* (leaf-beetles; including the seed beetles, Bruchidae), which mainly feed on green plant parts [4].

A widely accepted explanation for the great species diversity in beetles and other phytophagous insects is their co-diversification with the rapidly radiating land plants in the Tertiary [5]–[8]. In the Phytophaga, the phylogeny of beetle herbivores is thought to mirror that of major lineages of angiosperms, i.e. ancestral host associations in the Chrysomelidae reflect the available host plant lineages at that time (contemporaneous lineage diversification [3], [7], [9]–[12]). According to these studies, the most basal lineages of the Chrysomelidae appear associated with the primitive cycads (Aulacoscelidinae) and conifers (Palophaginae, Orsodacninae), followed by a large diversification of lineages on dicotyledoneous angiosperms (Chrysomelinae, Galerucinae, Cryptocephalinae) and monocots (some bruchids, Criocerinae, Donaciinae, Cassidinae and Hispinae). The association with monocots is thought to be primary, as the result of conservative host associations since the origin of the host in the Cretaceous [9], [12]. Ancient associations have also been proposed within the dicots, e.g. the co-radiation of the genus *Blepharida* (Alticinae) with the incense tree family Burseraceae dated to >100 Mya [13]. These scenarios would place the origin of all major lineages of chrysomelids well into the mid-Cretaceous. Recent phylogenetic studies of Chrysomelidae based on combined analyses of morphological data and 18S rRNA (SSU) sequences generally seem to confirm these conclusions, dating the origin of the Chrysomelidae to approximately 150–175 Mya [3], [11], [14].

However, the early-Cretaceous origin of Chrysomelidae is problematic because confirmed first body fossil records of all major subfamilies are known only from the Eocene (33.9–55.8 Mya), leaving a large gap between presumed origin and earliest appearance. Only a single fossil of a primitive chrysomelid of unclear subfamilial association has been dated to 72 Mya [15], [16]. Further, feeding damage to fossil leaves ascribed to hispine beetles (subfamily Cassidinae) has been dated to the earliest Cenozoic (65 Mya) and Eocene (52 Mya) [9], pre-dating Eocene body fossils of this group by some 20 My [9]. Even with this greater lineage age, a serious discrepancy remains between the fossil record and the much older molecular calibrations. As pointed out by Grimaldi and Engel [16], whilst the co-diversification hypothesis “makes great sense […] it will be very interesting to see if it is supported by future discoveries of fossils and by rigorous phylogenetic work”.

Here, we analyze basal relationships in Chrysomelidae independently of the morphological evidence that has been a major contribution in previous phylogenetic studies [3], [9]–[11]. We greatly expand taxon sampling for existing 18S rRNA (SSU) data and add two further (partial) ribosomal genes, mitochondrial 16S rRNA (*rrnL*) and nuclear 28S rRNA (LSU). The new tree is dated using fossils and a recent biogeographical estimate based on the Eocene split of Nearctic and Palearctic lineages of Timarchini [17]. These calibrations result in a younger age of major lineages than assumed by previous authors, and contradict the notion of an ancestral lineage association of angiosperms and phytophagous Chrysomelidae.

## Results

**Figure 1:**
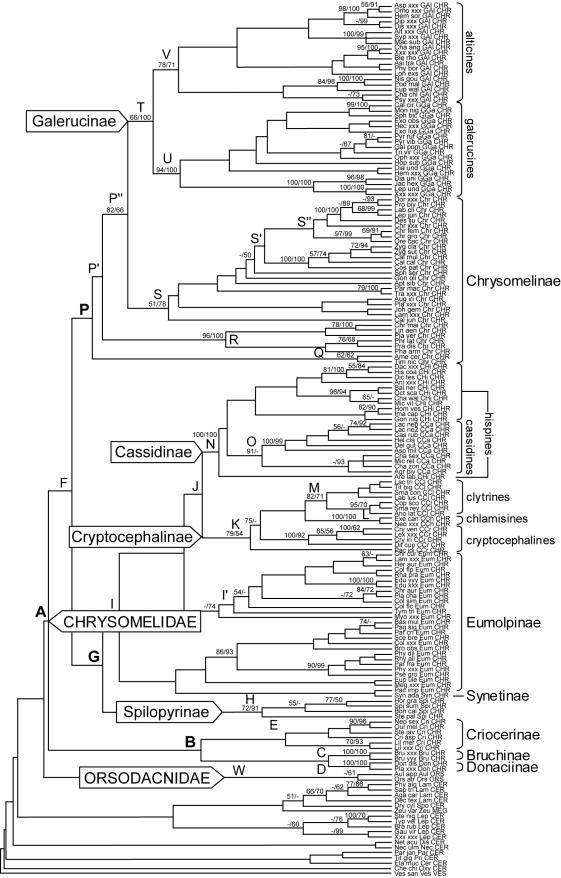
Most parsimonious tree for the Chrysomelidae based on *rrnL*, SSU and LSU ribosomal markers from direct optimization [44] under equal weighting (10,105 steps). Numbers above branches represent parsimony bootstrap support values above 50% using a matrix excluding all gapped positions and maximum likelihood boostrap support above 50%. Clades mentioned in the text are highlighted.

**Figure 2:**
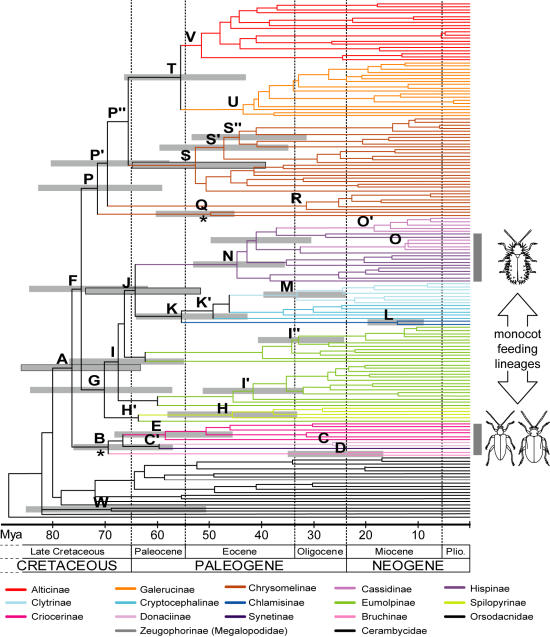
Maximum likelihood tree constrained for a molecular clock. This tree topology was obtained implementing a GTR+G+I evolutionary model in PHYML. The nodes used to calibrate the tree based on a sagrine-like fossil (72 Mya) and the vicariance of Timarchini (48 Mya) are marked with an asterisk. The average node age from both calibrations differs only slightly (5.4 My at the Chrysomelidae nodes) and hence the mean of both values was used for the figure. A gray bar represents the combined confidence interval from character resampling based on these two calibration points for several key nodes. Nodes for taxonomic groups of interest are labelled using the same key as in Figure 1.

Simultaneous analysis of the three length variable markers was performed using direct optimization under parsimony (Figure 1) and maximum likelihood (ML) analysis on an alignment from BLAST (Figure 2). Direct optimization under equal weighting of indels and nucleotide changes produced a shortest tree of 10,105 steps (CI = 0.332, RI = 0.607). BLAST-based alignments resulted in more condensed aligned data matrices (3304 versus 4579 sites in the implied alignment from direct optimization), but the differences mostly affected autapomorphic changes rather than parsimony informative and ungapped sites (Table 1). Analyses on the combined data produced very similar trees. The Chrysomelidae s. str. was monophyletic (node A), with Orsodacnidae as its sister group (W; parsimony only). Chrysomelidae appeared subdivided in three major clades, including the ‘sagrines’ (node B), ‘eumolpines’ (node G) and ‘chrysomelines’ (node P). The ‘sagrines’ included Bruchinae (C) and the two monocot feeding subfamilies Donaciinae (D) and Criocerinae (E), and presumably including the monocot feeding Sagrinae [3], [11], [18] not sampled here. The ML tree also placed the Synetinae sister to Bruchinae (C' in Figure 2). The ‘chrysomeline’ clade (P) included the Chrysomelinae (paraphyletic [R, S] with Timarchini [Q] at the base; their monophyly not rejected by a SH test) plus the reciprocally monophyletic (T) Galerucinae (U) and Alticinae (V) (Galerucinae s.l.) nested within it.

**Table 1:** Summary of tree statistics and major phylogenetic findings in the simultaneous analyses of ribosomal data of Chrysomelidae using parsimony (Direct Optimization, DO) and maximum likelihood (ML) tree reconstructions.

	DO	BLAST (ML)
*Tree statistics*		
Aligned sites	4579	3304
Variable sites^a^	2648 (484)	1357 (409)
Informative sites^a^	1279 (331)	921 (266)
parsimony tree length	10105	[10467]^b^
likelihood score	[45699.7872]^c^	35041.1587
CI	0.332	[0.158]^b^
RI	0.607	[0.484]^b^
*Phylogenetic conclusions* d		
Chrysomelidae	M (sister to Orsodacnidae)	M (sister to Cerambycidae)
‘sagrines’ (Don+Cri+Bru)	M	M (incl. Syn)
‘chrysomelines’+‘eumolpines’	M	M
‘eumolpines’ (Spi+Eum+Cry+Cas)	M (incl. Syn)	M
Cassidinae s.l./s.str.^e^	M/M	M/Po
‘chrysomelines’ (Tim+Chr+Gal)	M	M
Tim+Chr	Pa	Pa
monocot feeding Chrysomelidae	Po	Po

a Excluding gapped sites in brackets.

b Optimized under parsimony in PAUP*, for comparative purposes only.

c Optimized with PAUP* implementing the GTR+I+G model, for comparative purposes only.

d M: monophyletic; Pa: paraphyletic; Po: polyphyletic.

e The Cassidinae s.l. includes hispines (paraphyletic in our analyses) and cassidines.

Subfamily abbreviations: Don-Donaciinae, Cri-Criocerinae, Bru-Bruchinae, Syn-Synetinae, Spi-Spilopyrinae, Eum-Eumolpinae, Cry-Cryptocephalinae, Cas-Cassidinae, Tim-Timarchinae, Chr-Chrysomelinae, Gal-Galerucinae.

The ‘eumolpines’ included several subfamilies which have not been linked in the past. Spilopyrinae (H) was sister to Eumolpinae (I) which itself was paraphyletic with respect to Synetinae (parsimony) (Figure 1). Also nested within this group were (i) the paraphyletic Cryptocephalinae s.l. (K), with Chlamisinae (L) and Clytrinae (M) nested within; and (ii) the monocot feeding Cassidinae+Hispinae (N), the latter paraphyletic with respect to a monophyletic (parsimony) or polyphyletic (ML) Cassidinae (O; and O' in Figure 2). The placement of monocot feeding Cassidinae+Hispinae ( = Cassidinae s.l.) within Eumolpinae was strongly supported in all analyses. A SH-test strongly rejected their proposed monophyly with the monocot feeding clade of the sagrine group [3,9, but see 11].

**Figure 3:**
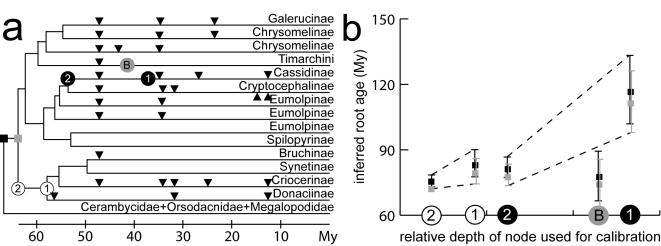
Dating the origin of Chrysomelidae under various absolute age calibrations. (a) Linearized tree with branch lengths proportional to substitution rate used to estimate the age of the Chrysomelidae. The uncertainty of fossil placements along the branches leading to a dated crown group is represented by the intervals (1, 2) (white: sagrine-like fossil; black: hispine-like feeding traces); the biogeographical event (“B” in gray circle) represents a maximum age for the vicariant split. Black triangles along branches represent the approximate placement of fossils known for each leaf beetle subfamily (all Eocenic or younger; Quaternary fossils excluded). (b) Inferred ages and 94% confidence intervals for the Chrysomelidae. The origin of Chrysomelidae is defined by the first separation of basal lineages within the Chrysomelidae (minimum age; gray square) and the node separating Chrysomelidae from other Chrysomeloidea (maximum age; black square). These node ages (y-axis) were estimated with each of the three calibration points from dated fossils and biogeography. The precise age of the calibration points (x-axis) is affected by uncertainty regarding their placement along the branch defining the crown group, bracketed by the interval (1, 2), and therefore a range of dates on the y-axis (origin of Chrysomelidae) is given for the upper and lower bounds of the calibrations. These calibration points each have a confidence interval from character resampling shown by error bars. The tree in (a) was scaled to match the minimum possible root age according to the analysis in (b), i.e. the lower boundary of the confidence interval using the biogeographical calibration.

The ML tree was constrained to a molecular clock and branch lengths were optimized using the penalized likelihood method [19] under an optimal smoothing parameter of 3.6. The tree was scaled for absolute ages by enforcing the oldest leaf beetle fossil ages to the relevant nodes as minimum ages, and by setting the split of Nearctic and Palearctic lineages of Timarchini to 48 Mya [17] (Figure 2). This places the origin of the Chrysomelidae s. str. at 79.2 Mya (paleontological dating; confidence interval 74.4–86.1 Mya) or 73.8 Mya (biogeographical dating; 63.7–85.6), and the separation of most subfamilies to a narrow time window between 73 and 55 Mya at the boundary of the Cretaceous and the Paleogene (Figure 2, Table 2 and Table S1). These estimates are concordant with the current fossil record for the Chrysomelidae (Figure 3). Older dates for the origin of Chrysomelidae were obtained with the use of (i) the calibration point for *Blepharida* dated based on the separation of Ethiopian and Neotropical subclades with the split of western Gondwana [13], and (ii) the hispine feeding traces on ginger leaves from North America [9]. The former was mapped on the current tree by setting the node separating the representative of *Blepharida* from its closest relative to 112 Mya (a conservative estimate, as the closest relative of *Blepharida* in our study was more distant than the genus *Diamphida* used in the original work). This resulted in a date for the origin of Chrysomelidae of 216 Mya (168.6–228.0). Similarly, calibrated with the younger of the two fossil feeding traces, the age of Chrysomelidae increased to 89.0 Mya (confidence interval 78.4–100.9), whereas the older traces pushed back their age to 111.3 Mya (97.9–126.1; Table 2). This calibration would set the Nearctic-Palearctic split of Timarchini to 72.4 Mya (59.8–87.5; Table 2), which is well before the continental separation and seems implausible.

**Table 2:** Dated events in the evolution of the Chrysomelidae using various calibration points for dating the phylogram in Figure 2.

		Biogeography (48 Mya)		“sagrine” fossil (72 Mya)		feeding traces (52 Mya)		feeding traces (65 Mya, low)		feeding traces (65 Mya, high)	
Lineage	Node	Age (Mya)	94% confidence interval	Age (Mya)	94% confidence interval	Age (Mya)	94% confidence interval	Age (Mya)	94% confidence interval	Age (Mya)	94% confidence interval
Chrysomelidae	A	73.2	63.1–84.8	79.2	74.4–86.1	89.0	78.4–100.9	77.5	73.7–83.5	111.3	97.9–126.1
‘sagrine’ clade	B	66.5	56.8–75.9	72.0	-	80.9	70.7–92.5	70.4	63.5–77.5	101.2	88.4–115.7
‘chrysomeline’+‘eumolpine’ clades	F	71.5	61.8–82.4	77.3	74.3–89.6	86.9	76.2–98.7	75.6	72.5–80.5	108.6	95.3–123.4
‘eumolpine’ clade	G	67.3	57.0–79.1	72.7	72.6–84.3	81.7	71.7–92.5	71.1	68.4–75.7	102.2	89.6–115.7
Cassidinae s.l.	N	42.8	35.4–51.7	46.3	40.5–53.0	52.0	-	45.3	40.7–51.1	65.0	-
oldest Cassidinae s.str.	O+O'	35.6	30.5–47.8	42.5	36.4–50.0	47.7	42.1–52.0	41.5	36.5–46.4	59.6	52.6–65.0
‘chrysomeline’ clade	P	68.5	58.8–78.0	74.2	67.8–80.6	83.4	73.1–96.6	72.6	67.3–79.5	104.3	91.4–120.7
North Atlantic vicariance *Timarcha*	Q	48.0	-	51.5	45.2–60.3	57.9	47.8–70.0	50.4	44.3–60.0	72.4	59.8–87.5

The age corresponds to the most recent common ancestor of the corresponding crown group. For the 65 Myo feeding traces the entire dating interval is given. For additional dated nodes and intervals see Table S1.

However, the placement of the hispine traces was problematic due to uncertainty about where precisely to fix them along the long branch leading to cassidines. Standard procedures for fossil calibrations [20] use the basal node of the crown group for the calibration (i.e., the fossil age is placed at the immediate ancestor to the extant lineage). In the case of the older feeding tracks, they can be set to 65 Mya at the base of the crown group resulting in the old age for the Chrysomelidae (black ‘1’ in Figure 3), but if the dates of the feeding traces are moved back towards the base of the long branch leading to hispine/cassidine, i.e. the earliest point these fossils could mark on the tree (black ‘2’ in Figure 3), this would place the origin of Chrysomelidae to 77.4 Mya (73.7–83.5), in agreement with all other estimates (Table 2). Equally, if the younger feeding traces are moved back to the base of this branch, the age estimate for Chrysomelidae is reduced to 62.0 Mya (59.0–66.8).

## Discussion

This study provided a first comprehensive phylogenetic analysis of Chrysomelidae based on molecular data alone. We used two different approaches to alignment (direct optimization and homology-extension alignment) and tree building (parsimony and ML), to illustrate the effect of very different data treatments. Both procedures resulted in similar trees (Figures 1 and 2; Table 1). Other types of analysis based on a range of alignment procedures and search algorithms also confirmed these results (Gómez-Zurita et al., submitted). These analyses also determined that any of the three markers separately performed worse than the simultaneous analysis when assessed based on the recovery of well established groups of subfamilies (Gómez-Zurita et al., submitted). This suggests that the amount of data is critical for conclusions about basal relationships in Chrysomelidae. Previous analyses based on the single SSU gene were likely insufficient and greatly affected by morphological data used in simultaneous analysis [3], [11], [14]. The results obtained here now provide the basis for a new classification of Chrysomelidae, to include three main groups preliminarily named as ‘sagrines’, ‘chrysomelines’ and ‘eumolpines’. Whereas the former two clades largely correspond to natural groupings recognized previously, the ‘eumolpines’ were surprising. The analyses suggest the paraphyly of Eumolpinae with respect to the subfamilies Cryptocephalinae, Chlamisinae and Clytrinae (the Cryptocephalinae s.l., frequently referred to as ‘Camptosoma’), and most notably the Cassidinae/Hispinae. This was particularly unexpected because it separates the latter from the other monocot feeding lineage (Donaciinae+Criocerinae+Sagrinae), but this result was strongly supported in the SH test.

Our analysis places the origin of extant leaf beetle subfamilies to the end of the Cretaceous and the late Paleocene (73 to 55 Mya) (Table 2 and Table S1; Figure 2). Hence the basal chrysomelid diversification would be substantially younger than the radiation of their hosts, arguing against the widely accepted hypothesis of co-diversification of deep angiosperm lineages and their beetle herbivores. A much earlier date for the origin of Chrysomelidae has been proposed in previous studies [3], [9], but the evidence for such early radiation is weak. First, descriptions of fossil chrysomelids [21] from the Jurassic (146–200 Mya) and Triassic (200–250 Mya; i.e. nearly as old as the oldest fossils of Coleoptera at about 265 Mya [16], [22]) suffer from poor fossil preservation, insufficient diagnostic characters for reliable grouping with Chrysomelidae, or uncertain fossil ages [4], [21], [23]. They are now considered to be untenable [16]. Further, chrysomelid fossils are essentially absent in the Cretaceous and most appear in the Eocene (34–56 Mya), representing most major subfamilies [21], [24]. The oldest clearly identifiable record is *Donacia wightoni* from the Canadian Paleocene (56–66 Mya) [21]. Slightly older are the recently discovered Canadian Mesozoic fossils dated to 72 Mya which have been identified as sagrine-like primitive chrysomelids [15]. They probably represent an early lineage which pre-dates the diversification of major extant sufamilies.

The dating of feeding damage characteristic of rolled-leaf hispines to a maximum of 65 Mya [9], interpreted as corroborating the great antiquity of Chrysomelidae, as we show here it is still in agreement with the younger age if it is assumed that the feeding traces were produced by a stem group of hispines. It is also conceivable that these feeding tracks were produced by groups other than hispine Cassidinae [16], perhaps due to convergent feeding patterns in other extant or extinct leaf feeding insect lineages [25]. But even if confirmed, they do not refute our calibration while being consistent with the dating of hispine body fossils to the middle Eocene [21]. These dates are, however, of importance for interpreting evolutionary history, e.g. in hispine *Cephaloleia* leaf rollers that were recently used to link speciation rate to paleoclimatic history in the Tertiary [12]. This study calibrated the basal *Cephaloleia* node with the feeding traces of 66.2 Mya (implying they were produced by an ancestor of extant *Cephaloleia*), but this date could equally have been ascribed to a stem lineage of *Cephaloleia* or other hispines basal to the *Cephaloleia* node, shifting the origin of this genus towards the present. Only the *Blepharida* calibration point [13] remains strongly inconsistent with the current estimate, but this date depends on a strict vicariance scenario for the separation of Afrotropical and Neotropical lineages in the genus despite evidence for long-distance dispersal between these insect faunas [e.g., 26] and hence a possible later origin of independent lineages on either continent. Most flea beetles, including *Blepharida*, are flighted and dispersive, in contrast to the flightless, sedentary *Timarcha* whose Nearctic-Palearctic separation is more likely to reflect ancient vicariance.

Unquestionable angiosperms in the fossil record of a magnoliid are from ca. 130 Mya [27], and the recent discovery of fossilized “flowers” of *Archaefructus* from China would possibly push back the origin of angiosperms even further. These fossils were found in the lower part of the Yixian formation dated to between 125 and 145 Mya [28], [29]. These authors' conservative estimate suggested a minimum age of 124.6 my [29], but possibly as ancient as the oldest deposits in the formation in which they were found, placing the origin of angiosperms to the Jurassic-Cretaceous boundary (142 Mya) [28]. Molecular calibrations and improved dating methods have converged on estimates between 140–180 Mya, predating by 10–50 My the dates inferred from the fossil record [e.g., 30]. The origin of monocot angiosperms has now been dated reliably to the Early Cretaceous based on molecular clock [31], [32] and fossil [33] evidence. Hence, by the time the Cassidinae/Hispinae originated, their host plants would have already been an ancient lineage that had widely diversified.

In addition, our rejection of the purported sister relationship of Cassidinae with the remaining monocot feeders of the ‘sagrine’ clade contradicts the proposed history of a single origin of monocot feeding in chrysomelids [9]. Instead, the evolution of host associations involved multiple colonizations of monocots, rather than the repeated secondary change to dicots from a primitively monocot feeding lineage (semi-aquatic monocots for the Donaciinae) postulated by [9]. Our study therefore adds to the growing number of cases showing a time lag between host radiation and the eventual colonization by insect herbivores [34], [35]. While the host plants represented an almost infinite diversity of suitable niches for chrysomelids, their clade diversification was not in parallel, but instead involved the expansion into an existing, much older resource that likely promoted specialization and speciation [36]. Hence the assertions of concurrent clade evolution of leaf beetles with their monocot and eudicot host lineages [3], [9] may have to be revised, and where their phylogenies are congruent this should be interpreted as the result of adaptive radiation rather than ancestral co-cladogenesis [e.g., 37]. Our findings do not argue against co-evolution as a potential driving force for speciation in particular subclades, but are clearly not compatible with the proposed co-evolution scenario since the time of the ancestor of the Chrysomelidae. It remains to be seen to what extent the conclusions about the ancient associations of basal Chrysomeloidea and Curculionoidea with their cicad and conifer feeding hosts [3] will be affected by the revised clock calibrations proposed here.

## Material and Methods

### Taxon Sampling and DNA Sequencing

We sampled all currently recognized subfamilies of Chrysomelidae, except for Sagrinae and Lamprosomatinae (Table S2). The former consistently has been found as sister to Bruchinae [11], [18], whereas Lamprosomatinae is likely associated to a lineage of Chlamisinae, Clytrinae and Cryptocephalinae [38]. We also sampled the families Orsodacnidae (including both subfamilies Orsodacninae and Aulacoscelidinae) and Megalopodidae, considered to be primitive Chrysomeloidea. The related Cerambycidae were represented by members of nine subfamilies [39], including all major groups. Trees were rooted using *Vesperus sanzi* (Vesperidae), a plesiomorphic group near or within Cerambycidae [40].

Total DNA was extracted from whole specimens or abdominal tissue in large specimens using the DNeasy Tissue kit (QIAGEN). Primer combinations, PCR conditions and PCR product purification were as in [41] for SSU and [42] for the other markers. PCR products were sequenced in both directions using the ABI (Applied Biosystems) technology. The sequences were deposited in GenBank under the accession numbers in Table S2.

### Phylogenetic Analyses

Homology assignment for the ribosomal data was carried out using (i) a ‘progressive’ alignment procedure as implemented in BlastAlign [43] for maximum likelihood (ML) searches using PHYML 2.4 [44], and (ii) a parsimony based sequence alignment applying the concept of dynamic homology [45], [46] in POY 3.0.11 [45], [47]. The latter involved tree searches in three consecutive stages of increasing computational intensity [48], conducted under equal costs for character changes and indels. Although the method estimates a tree directly from nucleotide variation and length differences, an ‘implied alignment’ can be obtained post-hoc for further searches and diagnostics [45], including ML analyses and bootstrap resampling.

PHYML likelihood searches were run under the evolutionary models and estimated parameters as obtained from ModelTest 3.06 [49] and starting from a tree obtained using the modified neighbor-joining algorithm BIONJ [50]. Node robustness was assessed using non-parametric bootstrapping and 100 pseudoreplicates. Tree searches were performed on a parallel processing system using 16 dual-processors (2.8GHz P4, 2GB RAM).

### Molecular Clock Calibration

Penalized likelihood [19] was implemented in the r8s v.1.71 software on the ML tree topology. The optimal value for the smoothing parameter which accounts for the roughness (rate change between branches) of the tree was obtained by cross-validation using 20 steps of successive increases of 0.2. Absolute ages were established based on the two oldest known leaf beetle fossils, namely a sagrine-like beetle in 72 Myo amber from Canada [15], [16] and 65 Myo feeding traces attributed to a hispine beetle [9]. These fossils are considered to provide minimum ages for the dated clades [20], because of uncertainty about the placement of the dated fossil within the crown group [35], [51]. However, these ‘minimum ages’ could also be placed along the branch leading up to the crown group if the fossil predates its earliest branching events, pushing their position back on the tree to the stem group age. The length of the branch leading to the crown group, and the resulting time interval to which a fossil can be placed, is in part dependent on sampling density of basal groups, as the addition of basal branches can push back the relevant crown group node (but never make it younger). So we calculated all ‘minimum age’ calibrations by placing the fossil data at the base and the tip of the focal branches, providing minimum and maximum ages, respectively, for the nodes in the phylogram. In addition, a biogeographic calibration was used applying a well-established vicariant split of western Palearctic *Timarcha* and Nearctic *Americanotimarcha* [52] with the Eocene (34–49 Mya; [53]) opening of the North Atlantic. This node was dated to approximately 48 Mya in a previous work [17]. Following [54], we estimated the 94% confidence intervals for clade ages given the stochastic error of rate variation along branches. This was done by bootstrapping the original data matrix 100 times with seqboot in PHYLIP v. 3.65 [55], optimizing branch lengths on the tree topology using PHYML, and linearizing the trees with r8s.

## Supporting Information

Table S1 Dated events in the evolution of the Chrysomelidae using various calibration points for dating the phylogram in Figure 2. The age corresponds to the most recent common ancestor of the corresponding crown group. For the 65 Myo feeding traces the entire dating interval is given.
(0.05 MB DOC)

Table S2 Taxon sampling, voucher and nucleotide database accession numbers. In all, we obtained sequences for 167 Chrysomeloidea including 147 representatives of Chrysomelidae from 146 species in 134 genera, plus 16 genera and species of Cerambycidae, two of Orsodacnidae, one each of Megalopodidae and Vesperidae.
(0.24 MB DOC)
