# Supplementary material for: Recalibrated Tree of Leaf Beetles (Chrysomelidae) Indicates Independent Diversification of Angiosperms and Their Insect Herbivores
Source: PLoS One. 2007 Apr 11;2(4):e360. doi: 10.1371/journal.pone.0000360 (PMC1832224; doi:10.1371/journal.pone.0000360)
Supplement: Table S1 — Dated events in the evolution of the Chrysomelidae using various calibration points for dating the phylogram in Figure 2. The age corresponds to the most recent common ancestor of the corresponding crown group. For the 65 Myo feeding traces the entire dating interval is given. (0.05 MB DOC) [file pone.0000360.s001.doc]

**Table S1.** Dated events in the evolution of the Chrysomelidae using various calibration points for dating the phylogram in Figure 2. The age corresponds to the most recent common ancestor of the corresponding crown group. For the 65 Myo feeding traces the entire dating interval is given.

|  |  | **Biogeography** (48 Mya) | | **“sagrine” fossil**  **(72 Mya)** | | **feeding traces**  **(52 Mya)** | | **feeding traces**  **(65 Mya, low)** | | **feeding traces**  **(65 Mya, high)** | |
| --- | --- | --- | --- | --- | --- | --- | --- | --- | --- | --- | --- |
| Lineage | Node | **Age**  **(Mya)** | **94%**  **confidence**  **interval** | **Age**  **(Mya)** | **94%**  **confidence**  **interval** | **Age**  **(Mya)** | **94%**  **confidence**  **interval** | **Age**  **(Mya)** | **94%**  **confidence**  **interval** | **Age**  **(Mya)** | **94%**  **confidence**  **interval** |
| Orsodacnidae | W | 65.9 | 50.5-80.8 | 71.4 | 59.9-85.1 | 80.3 | 65.4-99.5 | 69.8 | 57.5-88.0 | 100.3 | 81.8-124.4 |
| Donacia+Plateumaris | D | 25.2 | 16.7-33.2 | 27.2 | 20.1-35.2 | 30.6 | 22.1-40.2 | 26.6 | 20.0-34.8 | 38.2 | 27.6-50.2 |
| Criocerinae | E | 55.9 | 45.5-67.9 | 60.5 | 53.6-68.2 | 68.0 | 58.8-81-5 | 59.2 | 51.5-68.9 | 85.0 | 73.5-101.8 |
| Spilopyrinae | H | 43.7 | 33.4-54.2 | 47.4 | 39.0-58.3 | 53.3 | 41.9-65.1 | 46.4 | 37.8-58.3 | 66.6 | 52.4-81.3 |
| Eumolpinae (without *Eupales*) | I | 64.7 | 54.7-75.5 | 70.0 | 64.3-76.7 | 78.7 | 69.6-89.7 | 68.5 | 65.7-73.1 | 98.4 | 87.0-112.1 |
| Eumolpini | I’ | 39.8 | 32.1-49.3 | 43.0 | 37.3-51.5 | 48.4 | 40.4-59.6 | 42.1 | 36.6-50.9 | 60.5 | 50.5-74.5 |
| Typophorini | I’’ | 32.8 | 24.1-39.3 | 34.0 | 27.9-40.8 | 38,3 | 30.7-47.0 | 33.3 | 28.3-38.8 | 47.8 | 38.4-58.7 |
| Cryptocephalinae s.l./Cassidinae s.l. | J | 61.5 | 51.6-69.8 | 66.5 | 60.4-73.7 | 74.7 | 66,2.83.1 | 65.0 | - | 93.4 | 82.8-103.9 |
| Cryptocephalinae s.l. | K | 53.4 | 42.7-63.5 | 57.3 | 49.0-63.9 | 64.4 | 54.9-73.4 | 56.1 | 51.0-61.2 | 80.5 | 68.6-91.8 |
| Chlamisinae | L | 13.4 | 8.9-18.1 | 14.5 | 10.2-19.8 | 16.3 | 11.0-21.8 | 14.2 | 10.1-19.0 | 20.4 | 13.8-27.2 |
| Clytrinae s.str. | M | 31.7 | 23.6-37.4 | 34.2 | 26.9-39.4 | 38.4 | 30.1-46.2 | 33.4 | 26.4-37.9 | 48.0 | 37.6-57.7 |
| Chrysomelini | P’ | 66.7 | 57.6-76.0 | 72.2 | 66.8-80.4 | 81.2 | 71.4-95.0 | 70.6 | 66.1-78.7 | 101.5 | 89.2-118.7 |
| “modern” Chrysomelinae | S | 50.0 | 39.2-60.6 | 54.5 | 45.6-64.8 | 61.2 | 47.4-71.1 | 53.3 | 43.9-61.5 | 76.5 | 59.2-88.9 |
| Doryphorina+Chrysolinina | S’ | 45.2 | 34.8-54.8 | 48.9 | 38.4-59.8 | 55.0 | 41.5-65.0 | 47.9 | 38.8-57.3 | 68.7 | 51.8-81.2 |
| Chrysolinina | S’’ | 42.9 | 31.4-51.5 | 46.0 | 35.0-53.7 | 51.7 | 39.1-59.9 | 45.0 | 35.3-52.7 | 64.6 | 48.9-74.9 |
| Galerucinae s.l. | T | 53.2 | 43.1-62.1 | 57.5 | 50.4-66.5 | 64.6 | 55.0-77.6 | 56.2 | 50.0-64.2 | 80.8 | 68.7-97.0 |
