# Supplementary material for: Recalibrated Tree of Leaf Beetles (Chrysomelidae) Indicates Independent Diversification of Angiosperms and Their Insect Herbivores
Source: PLoS One. 2007 Apr 11;2(4):e360. doi: 10.1371/journal.pone.0000360 (PMC1832224; doi:10.1371/journal.pone.0000360)
Supplement: Table S2 — Taxon sampling, voucher and nucleotide database accession numbers. In all, we obtained sequences for 167 Chrysomeloidea including 147 representatives of Chrysomelidae from 146 species in 134 genera, plus 16 genera and species of Cerambycidae, two of Orsodacnidae, one each of Megalopodidae and Vesperidae. (0.24 MB DOC) [file pone.0000360.s002.doc]

**Table S2.** Taxon sampling, voucher and nucleotide database accession numbers. In all, we obtained sequences for 167 Chrysomeloidea including 147 representatives of Chrysomelidae from 146 species in 134 genera, plus 16 genera and species of Cerambycidae, two of Orsodacnidae, one each of Megalopodidae and Vesperidae.

| Family  Subfamily  Tribe | Species | BM Acc.  No. | Code | Locality | *rrnL* | SSU | LSU |
| --- | --- | --- | --- | --- | --- | --- | --- |
| CERAMBYCIDAE |  |  |  |  |  |  |  |
| Cerambycinae | *Elaphidion mucronatus* (Say) | BM704354 | ElamucCerCER | USA: NJ, Atlantic Co., Linwood | AJ841404 | AJ841525 | AJ841654 |
| Disteniinae | *Nethinius acuticollis* Villiers | - | NetacuDisCER | Madagascar: Masoala | - | AJ841526 | AJ841655 |
| Lamiinae | *Agapanthia cardui* (Linnaeus) | BM704355 | AgacarLamCER | Spain: Madrid, El Pardo | AJ841405 | AJ841527 | AJ841656 |
|  | *Dectes texanus* LeConte | BM704358 | DectexLamCER | USA: VA, Rockingham Co., Harrisonburg | AJ841406 | AJ841528 | AJ841657 |
|  | *Phytoecia algerica* Desbrochers | BM704356 | PhyalgLamCER | Spain: Segovia, Lastras de Cuéllar | AJ841407 | AJ841529 | AJ841658 |
|  | *Saperda tridentata* Olivier | BM704357 | SaptriLamCER | USA: VA, Shenandoa Co., Edinburg | AJ841408 | AJ841530 | AJ841659 |
| Lepturinae | *Brachyleptura rubrica* (Say) | BM704362 | BrarubLepCER | USA: NJ, Cape May Co., Cape May Point | AJ841409 | AJ841531 | AJ841660 |
|  | *Gaurotes virginea* (Linnaeus) | BM704359 | GauvirLepCER | Germany: Benediktbeuern | - | AJ841532 | AJ841661 |
|  | *Stenurella nigra* (Linnaeus) | BM704360 | StenigLepCER | Spain: Madrid, El Pardo | - | AJ841533 | AJ841662 |
|  | *Typocerus velutina* (Olivier) | BM704363 | TypvelLepCER | USA: WV, Greenbrier Co., Tuckahoe Lake | AJ841410 | AJ841534 | AJ841663 |
|  | Lepturinae sp. JGZ | BM704361 | XxxxxxLepCER | USA: NJ, Cape May Co., Cape May Point | AJ841411 | AJ841535 | AJ841664 |
| Necydalinae | *Necydalis ulmi* Chevrolat | BM704364 | NeculmNecCER | France: Les Mayons | AJ841412 | AJ841536 | AJ841665 |
| Oxypeltinae | *Cheloderus childreni* (Blanchard) | - | ChechiOxyCER | Chile: Talca, Altos de Vilches | AJ841413 | AJ841537 | AJ841666 |
| Parandrinae | *Parandra janus* Bates | - | ParjanParCER | Malaysia: Sabah, Trus Madi | - | AJ841538 | AJ841667 |
| Prioninae | *Titanus giganteus* (Linnaeus) | - | TitgigPriCER | French Guiana: Montagnes Tresor, Camp Caiman | AJ841414 | AJ841539 | AJ841668 |
| Spondylidiinae | *Drymochares cylindraceus* (Fairmaire) | - | DrycylSpoCER | Spain: Salamanca, Sierra de Candelario | - | AJ841540 | AJ841669 |
| CHRYSOMELIDAE |  |  |  |  |  |  |  |
| Bruchinae | *Bruchidius sp.* 1 | BM704365 | BruxxxBruCHR | Spain: Ávila, Aveinte | AJ841299 | AJ841415 | AJ841542 |
|  | *Bruchidius sp.* 2 | BM704366 | BruyyyBruCHR | Spain: Badajoz, Garbayuela | AJ781557 | AJ781620 | AJ781683 |
| Chrysomelinae |  |  |  |  |  |  |  |
| Chrysomelini | *Apterocuris sibirica* (Gebler) | BM704367 | AptsibChrCHR | Kazakhstan: Ivanovsky Mt. Range, Prohodnoi Belok mts. | AJ841300 | AJ841416 | AJ841543 |
|  | *Augomela iridea* Baly | BM704368 | AugiriChrCHR | Australia: QLD, Brisbane, Mt. Coot-tha | AJ841301 | AJ841417 | AJ841544 |
|  | *Calligrapha californica coreopsivora* Brown | BM704369 | CalcalChrCHR | Canada: Ontario, Lanark Co., Almonte | AJ841302 | AJ841418 | AJ841545 |
|  | *Calligrapha multipunctata bigsbyana* (Kirby) | BM704370 | CalmulChrCHR | Canada: Quebec, L’Outaouais, Aylmer | AJ841303 | AJ841419 | AJ841546 |
|  | *Calomela juncta* Lea | BM704371 | CaljunChrCHR | Australia: QLD, Brisbane, McGregor Pk., Sunny Bank | AJ841304 | AJ841420 | AJ841547 |
|  | *Chrysolina femoralis* (Olivier) | BM704372 | ChrfemChrCHR | Spain: Segovia, Cuéllar | AJ841305 | AJ841421 | AJ841548 |
|  | *Chrysolina grossa* (Fabricius) | BM704373 | ChrgroChrCHR | Morocco: Jbel Tazerca | AJ841306 | AJ841422 | AJ841549 |
|  | *Chrysolina (Atechna) sp.* | BM704396 | ChrxxxChrCHR | South Africa: KZN, Eshowe | AJ841327 | AJ841444 | AJ841571 |
|  | *Chrysomela mainensis* Bechyné | BM704374 | ChrmaiChrCHR | Canada: Quebec, L’Outaouais, Quyon | AJ841307 | AJ841423 | AJ841550 |
|  | *Cosmogramma patricia* Erichson | BM704395 | CospatChrCHR | Bolivia: Cochabamba, km 102 to Villa Tunari | AJ841326 | AJ841443 | AJ841570 |
|  | *Desmogramma ljunghi* Stål | BM704375 | DesljuChrCHR | Brazil: Paraiba, Areia Branca | AJ841308 | AJ841424 | AJ841551 |
|  | *Doryphora sp.* | BM704376 | DorxxxChrCHR | Brazil: Betania, Colombo | AJ841309 | AJ841425 | AJ841552 |
|  | *Gonioctena olivacea* (Forster) | BM704377 | GonoliChrCHR | UK: Surrey, Sunningdale, Chobbam Common | AJ841310 | AJ841426 | AJ841553 |
|  | *Johannica gemellata* (Westwood) | BM704378 | JohgemChrCHR | Australia: QLD, Lamington N.P., Gwongoorool Pool | AJ841311 | AJ841427 | AJ841554 |
|  | *Labidomera clivicollis* Kirby | BM704379 | LabcliChrCHR | Canada: Quebec, L’Outaouais, Quyon | AJ841312 | AJ841428 | AJ841555 |
|  | *Lamprolina sp.* | BM704380 | LamxxxChrCHR | Australia: QLD, Brisbane, Mt. Coot-tha | AJ841313 | AJ841429 | AJ841556 |
|  | *Leptinotarsa juncta* (Germar) | BM704381 | LepjunChrCHR | USA: WV, Randolph Co., Elkwater | AJ841314 | AJ841430 | AJ841557 |
|  | *Linaeidea aenea* (Linnaeus) | BM704382 | LinaenChrCHR | Germany: Benediktbeuern | AJ781556 | AJ781619 | AJ781682 |
|  | *Oreina cacaliae* (Schrank) | BM704383 | OrecacChrCHR | Germany: Bavaria, Oberammergau, Lain Valley | AJ841315 | AJ841431 | AJ841558 |
|  | *Paropsis maculata* (Marsham) | BM704384 | ParmacChrCHR | Australia: QLD, Brisbane, Mt. Coot-tha | AJ841316 | AJ841432 | AJ841559 |
|  | *Phaedon armoraciae* (Linnaeus) | BM704385 | PhaarmChrCHR | UK: Surrey, Dorking, Ranmore Common | AJ841317 | AJ841433 | AJ841560 |
|  | *Phratora laticollis* Suffrian | BM704386 | PhrlatChrCHR | UK: London, NHM Wildlife Gardens | AJ841318 | AJ841434 | AJ841561 |
|  | *Plagiodera versicolora* (Laicharting) | BM704387 | PlaverChrCHR | Canada: Ontario, Lanark Co., Almonte | AJ841319 | AJ841435 | AJ841562 |
|  | *Plagiodera sp.* | BM704394 | PlaxxxChrCHR | Belize: Cayo, Las Cuevas Research Station | AJ841325 | AJ841442 | AJ841569 |
|  | *Prasocuris distincta* (Lucas) | BM704388 | PradisChrCHR | Tunisia: Rd Beja-Teboursouk Oued, 3 km NW Teboursouk | AJ841320 | AJ841436 | AJ841563 |
|  | *Proseicela bivittata* (Fabricius) | BM704389 | ProbivChrCHR | French Guiana: Cayenne Harbour, Mt. Rorota | - | AJ841437 | AJ841564 |
|  | *Sphaeratrix sericea* (Bechyné) | BM704390 | SphserChrCHR | South Africa: Kruger Park, Punda Maria | AJ841321 | AJ841438 | AJ841565 |
|  | *Trachymela sp.* | BM704391 | TraxxxChrCHR | Australia: QLD, Brisbane, McGregor Pk., Sunnybank | AJ841322 | AJ841439 | AJ841566 |
|  | *Zygogramma clathrata* Jacoby | BM704392 | ZygclaChrCHR | Mexico: Guerrero, Mezcala Bridge | AJ841323 | AJ841440 | AJ841567 |
|  | *Zygogramma suturalis* (Fabricius) | BM704393 | ZygsutChrCHR | USA: WV, Mason Co., Bellmeade | AJ841324 | AJ841441 | AJ841568 |
| Timarchini | *Americanotimarcha cerdo* Stål | BM704397 | AmecerChrCHR | USA: OR, Waldport | AJ278898 | AJ622054 | AJ841572 |
|  | *Timarcha nicaeensis* Villa | BM704398 | TimnicChrCHR | Italy: Salerno, Capo Palinuro, Cilento Natl. Pk. | AJ622036 | AJ622056 | AJ841573 |
| Criocerinae | *Crioceris asparagi* (Linnaeus) | BM704399 | CriaspCriCHR | USA: VA, Rockingham Co., Harrisonburg | AJ781558 | AJ781621 | AJ781684 |
|  | *Lilioceris merdigera* (Linnaeus) | BM704400 | LilmerCriCHR | Germany: Bavaria, Oberammergau, Lain Valley | AJ841328 | AJ841445 | AJ841574 |
|  | *Lilioceris sp.* | BM704401 | LilxxxCriCHR | Malaysia: Sabah, ca. 25 km SE Sapulut, Batu Punggul env. | AJ841329 | AJ841446 | AJ841575 |
|  | *Neolema sexpunctata* (Olivier) | BM704402 | NeosexCriCHR | USA: NJ, Monmouth Co., Roosevelt | AJ841330 | AJ841447 | AJ841576 |
|  | *Oulema melanopus* (Linnaeus) | BM704403 | OulmelCriCHR | Spain: Ávila, Solana de Rioalmar | AJ841331 | AJ841448 | AJ841577 |
|  | *Stethopachys javeti* Baly | BM704404 | StejavCriCHR | New Caledonia: South prov., Mt. Humboldt | AJ841332 | AJ841449 | AJ841578 |
| Cryptocephalinae (=Camptosoma) |  |  |  |  |  |  |  |
| Chlamisini | *Exema canadensis* Pierce | BM704405 | ExecanCChCHR | USA: OH, Lucas Co., Maumee Bay S. P. | AJ841333 | AJ841450 | AJ841579 |
|  | *Neochlamisus sp.* | BM704406 | NeoxxxCChCHR | USA: NJ, Monmouth Co., Roosevelt | AJ841334 | AJ841451 | AJ841580 |
| Clytrini | *Anomoea laticlavia* (Forster) | BM704407 | AnolatCClCHR | USA: NJ, Monmouth Co., Roosevelt | AJ841335 | AJ841452 | AJ841581 |
|  | *Coptocephala scopolina* (Linnaeus) | BM704408 | CopscoCClCHR | Spain: Salamanca, Buenamadre | AJ841336 | AJ841453 | AJ841582 |
|  | *Labidostomis lusitanica* (Germar) | BM704409 | LablusCClCHR | Spain: Soria, Embalse de la Cuerda del Pozo | AJ841337 | AJ841454 | AJ841583 |
|  | *Lachnaia tristigma* (Lacordaire) | BM704410 | LactriCClCHR | Spain: Salamanca, Buenamadre | AJ841338 | AJ841455 | AJ841584 |
|  | *Smaragdina concolor* Fabricius | BM704411 | SmaconCClCHR | Spain: Madrid, Batres | AJ841339 | AJ841456 | AJ841585 |
|  | *Smaragdina reyi* (Brisout) | BM704412 | SmareyCClCHR | Portugal: Viana do Castelo, Lovelhe | AJ841340 | AJ841457 | AJ841586 |
|  | *Tituboea biguttata* (Olivier) | BM704413 | TitbigCClCHR | Spain: Salamanca, Buenamadre | AJ841341 | AJ841458 | AJ841587 |
| Cryptocephalini | *Cryptocephalus iridipennis* Chapuis | BM704414 | CryiriCCrCHR | Australia: QLD, Brisbane, Mt. Coot-tha | AJ841342 | AJ841459 | AJ841588 |
|  | *Cryptocephalus venustus* Fabricius | BM704415 | CryvenCCrCHR | Canada: Quebec, L’Outaouais, Quyon | AJ841343 | AJ841460 | AJ841589 |
|  | *Ditropidus ?cupreus* Chapuis | BM704416 | DitcupCCrCHR | Australia: QLD, Brisbane, Mt. Coot-tha | AJ841344 | AJ841461 | AJ841590 |
|  | *Lexiphanes sp.* | BM704417 | LexxxxCCrCHR | French Guiana: Saint- Laurent du Maroni | AJ841345 | AJ841462 | AJ841591 |
|  | *Pachybrachis idiota* Suffrian | BM704418 | PacidiCCrCHR | French Guiana: Tresor Mountains, Road to Kaw | AJ841346 | AJ841463 | AJ841592 |
| Donaciinae | *Donacia distincta* LeConte | BM704419 | DondisDonCHR | Canada: Ontario, La Mer Bleue | AJ781559 | AJ781622 | AJ781685 |
|  | *Plateumaris sp.* | BM704420 | PlaxxxDonCHR | Canada: Quebec, L’Outaouais, Quyon | AJ841347 | AJ841464 | AJ841593 |
| Eumolpinae | *Basilepta multicostata* Jacoby | BM704421 | BasmulEumCHR | Malaysia: Sabah, ca. 25 km SE Sapulut, Batu Punggul env. | AJ781514 | AJ781568 | AJ781631 |
|  | *Bromius obscurus* (Linnaeus) | BM704422 | BroobsEumCHR | Russia: Tver region, Udomlya district, Kulikovo | AJ781551 | AJ781614 | AJ781677 |
|  | *Chrysochus auratus* (Fabricius) | BM704423 | ChraurEumCHR | USA: NJ, Monmouth Co., Roosevelt | AJ781546 | AJ781608 | AJ781671 |
|  | *Chrysodinopsis curtula* (Jacoby) | BM704424 | ChrcurEumCHR | Mexico: Guerrero, Mezcala Bridge | AJ781530 | AJ781590 | AJ781653 |
|  | *Colaspis gr. flavicornis* Fabricius | BM704425 | ColflcEumCHR | French Guiana: Cayenne Harbour, Mt. Rorota | AJ781531 | AJ781591 | AJ781654 |
|  | *Colaspis flavipes* (Olivier) | BM704426 | ColflpEumCHR | French Guiana: Cayenne Harbour, Mt. Rorota | AJ781532 | AJ781592 | AJ781655 |
|  | *Colaspoides nr. simillima* Baly | BM704427 | ColsimEumCHR | Malaysia: Pahang, Kuala Lipis env., Kg. Malaka env. | AJ781548 | AJ781610 | AJ781673 |
|  | *Colasposoma sp.* | BM704428 | ColxxxEumCHR | Malaysia: Pahang, Kuala Lipis env., Kenong Pumba Pk. | AJ781529 | AJ781587 | AJ781650 |
|  | *Edusella sp.* | BM704429 | EduyyyEumCHR | Australia: QLD, Brisbane, Mt. Coot-tha | AJ781543 | AJ781605 | AJ781668 |
|  | *Edusella sp.* | BM704430 | EduxxxEumCHR | Australia: QLD, Brisbane, Mt. Coot-tha | AJ781544 | AJ781606 | AJ781669 |
|  | *Eupales ulema* (Germar) | - | EupuleEumCHR | Greece: Ipiros, Ioanina, env. Papingo | AJ781511 | AJ781565 | AJ781628 |
|  | *Hermesia aurata* (Olivier) | BM704431 | HeraurEumCHR | French Guiana: Cayenne Harbour, Mt. Rorota | AJ781534 | AJ781595 | AJ781658 |
|  | *Lamprosphaerus sp.* | BM704432 | LamxxxEumCHR | French Guiana: Cayenne Harbour, Mt. Rorota | AJ781535 | AJ781596 | AJ781659 |
|  | *Megascelis sp.* | BM704433 | MegxxxEumCHR | Nicaragua: Managua, Ticuantepe, R.N.P. Montibelli | AJ781513 | AJ781567 | AJ781630 |
|  | *Myochrous sp.* | BM704434 | MyoxxxEumCHR | French Guiana: 30 km S Cayenne | AJ781552 | AJ781615 | AJ781678 |
|  | *Pachnephorus impressus* Rosenhauer | BM704435 | PacimpEumCHR | India: Chattisgarh, Durg, Bhilai Steel Township, Sector 8 Pk. | AJ781553 | AJ781616 | AJ781679 |
|  | *Pagria signata* (Motschulsky) | BM704436 | PagsigEumCHR | India: Chattisgarh, Durg, Bhilai Steel Township, Sector 8 Pk. | AJ781518 | AJ781572 | AJ781635 |
|  | *Parascela cribrata* (Schaufuss) | BM704437 | ParcriEumCHR | China: Hong Kong Is., Tai Tam Reg. Pk. | AJ781554 | AJ781617 | AJ781680 |
|  | *Paria fragariae* Wilcox | BM704438 | ParfraEumCHR | Canada: Ontario, Haldimand-Norfolk, Port Ryesse | AJ781521 | AJ781577 | AJ781640 |
|  | *Phytorus dilatatus* Jacoby | BM704440 | PhydilEumCHR | Malaysia: Sabah, ca. 25 km SE Sapulut, Batu Punggul env. | AJ781524 | AJ781580 | AJ781643 |
|  | *Phytorus sp.* | BM704441 | PhyxxxEumCHR | Indonesia: Sumatra, Gn Talamau, Ophir Mts., Simpangempat | AJ781525 | AJ781581 | AJ781644 |
|  | *Platycorynus chalybaeus* (Marshall) | BM704439 | PlachaEumCHR | Indonesia: Sumatra, Gn Talamau, Ophir Mts., Simpangempat | AJ781547 | AJ781609 | AJ781672 |
|  | *Pseudosyagrus grossepunctatus* Fairmaire | - | PsegroEumCHR | Madagascar: Amparihibe | AJ781526 | AJ781582 | AJ781645 |
|  | *Rhabdopterus praetextus* (Say) | BM704442 | RhapraEumCHR | Canada: Quebec, Quyon | AJ781541 | AJ781602 | AJ781665 |
|  | *Rhyparida alleni* Lea | BM704443 | RhyallEumCHR | Australia: QLD, Brisbane, Mt. Coot-tha | AJ781519 | AJ781573 | AJ781636 |
|  | *Scelodonta brevipilis* Lea | BM704444 | ScebreEumCHR | Australia: QLD, Brisbane, Mt. Coot-tha | AJ781550 | AJ781612 | AJ781675 |
|  | *Tymnes tricolor* (Fabricius) | BM704445 | TymtriEumCHR | USA: LA, W. Feliciana Pk., Feliciana Preserve | AJ781545 | AJ781607 | AJ781670 |
| Galerucinae (=Trichostoma) |  |  |  |  |  |  |  |
| Alticini | *Altica sp.* | BM704446 | AltxxxGAlCHR | Canada: Ontario, Kemptville | - | AJ841465 | AJ841594 |
|  | *Asiorestia transversa* (Marsham) | BM704447 | AsitraGAlCHR | Portugal: Viana do Castelo, Lovelhe | AJ841348 | AJ841466 | AJ841595 |
|  | *Asphaera sp.* | BM704448 | AspxxxGAlCHR | French Guiana: Tresor Mountains, Road to Kaw | AJ841349 | AJ841467 | AJ841596 |
|  | *Blepharida rhois* (Forster) | BM704449 | BlerhoGAlCHR | USA: NJ, Cape May Co., Cape May Point | AJ841350 | AJ841468 | AJ841597 |
|  | *Chabria ¿angulicollis* (Clark) | BM704450 | ChaangGAlCHR | Malaysia: Pahang, Kuala Lipis env., Kg. Malaka env. | AJ841351 | AJ841469 | AJ841598 |
|  | *Chaetocnema chlorophana* (Duftschmid) | BM704451 | ChachlGAlCHR | Spain: Ávila, Aveinte | - | AJ841470 | AJ841599 |
|  | *¿Diphaltica sp.* | BM704453 | DipxxxGAlCHR | French Guiana: Tresor Mountains, Road to Kaw, Camp Caiman | AJ841352 | AJ841471 | AJ841600 |
|  | *Disonycha sp.* | BM704454 | DisxxxGAlCHR | USA: OH, Pike Co., Jackson Lake | AJ841353 | AJ841472 | AJ841601 |
|  | *Euphitrea wallacei* Baly | BM704455 | EupwalGAlCHR | Malaysia: Sabah, ca. 25 km SE Sapulut, Batu Punggul env. | AJ841354 | AJ841473 | AJ841602 |
|  | *Hemipyxis nr. soror* Weise | - | HemsorGAlCHR | Madagascar: Anjanaharibe | AJ841355 | AJ841474 | AJ841603 |
|  | *Longitarsus exsoletus* (Linnaeus) | BM704456 | LonexsGAlCHR | Portugal: Viana do Castelo, Lovelhe | AJ841356 | AJ841475 | AJ841604 |
|  | *Macrohaltica subplicata* LeConte | BM704457 | MacsubGAlCHR | USA: OH, Pike Co., Jackson Lake | AJ841357 | AJ841476 | AJ841605 |
|  | *Nisotra goudoti* Harold |  | NisgouGAlCHR | Madagascar: Anjanaharibe | AJ841358 | AJ841477 | AJ841606 |
|  | *Omophoita sp.* | BM704458 | OmoxxxGAlCHR | French Guiana: So. Cayenne, crossroad N1-D5 | AJ841359 | AJ841478 | AJ841607 |
|  | *Phygasoma nr. borneonense* Jacoby | BM704459 | PhyborGAlCHR | Malaysia: Sabah, ca. 25 km SE Sapulut, Batu Punggul env. | AJ841360 | AJ841479 | AJ841608 |
|  | *Podagrica malvae* (Illiger) | BM704460 | PodmalGAlCHR | Spain: Madrid, Batres | AJ841361 | AJ841480 | AJ841609 |
|  | *Psylliodes sp.* | BM704452 | PsyxxxxGAlCHR | Spain: Madrid, Batres | AJ841362 | AJ841481 | AJ841610 |
|  | *Syphrea sp.* | BM704461 | SypxxxGAlCHR | French Guiana: Tresor Mountains, Road to Kaw | AJ841363 | AJ841482 | AJ841611 |
|  | Alticinae sp. | BM704462 | XxxxxxGAlCHR | Malaysia: Pahang, Cameron Highlands, Tahah Rata env. | AJ841364 | AJ841483 | AJ841612 |
| Galerucini | *Calomicrus circumfusus* (Marsham) | BM704463 | CalcirGGaCHR | Spain: Ávila, S. de Ávila, Pto. de las Fuentes | AJ841365 | AJ841484 | AJ841613 |
|  | *Diabrotica undecimpunctata howardi* Barber | BM704464 | DiaundGGaCHR | USA: VA, Rockingham Co., Harrisonburg | AJ781555 | AJ781618 | AJ781681 |
|  | *Diacantha unifasciata* (Olivier) | - | DiauniGGaCHR | Madagascar: Amparihibe | AJ841366 | AJ841485 | AJ841614 |
|  | *Exora obsoleta* (Fabricius) | BM704465 | ExoobsGGaCHR | Costa Rica: Heredia | AJ841367 | AJ841486 | AJ841615 |
|  | *Exosoma lusitanicum* (Linnaeus) | BM704466 | ExolusGGaCHR | Spain: Madrid, El Pardo | AJ841368 | AJ841487 | AJ841616 |
|  | *Galeruca pomonae* (Scopoli) | BM704467 | GalpomGGaCHR | Spain: Ciudad Real, Embalse de Peñarroya | AJ841369 | AJ841488 | AJ841617 |
|  | *?Hecataeus sp.* | BM704468 | HecxxxGGaCHR | French Guiana: So. Cayenne, crossroad N1-D5 | AJ841370 | AJ841489 | AJ841618 |
|  | *?Hemistus sp.* | BM704469 | HemxxxGGaCHR | Malaysia: Sabah, ca. 25 km SE Sapulut, Batu Punggul env. | AJ841371 | AJ841490 | AJ841619 |
|  | *Hoplosaenidea ?subcostata* (Jacoby) | BM704470 | HopsubGGaCHR | Malaysia: Sabah, ca. 25 km SE Sapulut, Batu Punggul env. | AJ841372 | AJ841491 | AJ841620 |
|  | *Jacobyanella hexaspilota* (Fairmaire) | - | JachexGGaCHR | Madagascar: Amparihibe | AJ841373 | AJ841492 | AJ841621 |
|  | *Leptaulaca undecimpunctata* (Klug) | - | LepundGGaCHR | Madagascar: Amparihibe | AJ841374 | AJ841493 | AJ841622 |
|  | *Monolepta nigripes* (Olivier) | BM704471 | MonnigGGaCHR | Malaysia: Sabah, Keningan env., Taman Bandukan | AJ841375 | AJ841494 | AJ841623 |
|  | *Ophraella sp.* | BM704472 | OphxxxGGaCHR | USA: OH, Pike Co., Jackson Lake | AJ841376 | AJ841495 | AJ841624 |
|  | *Pyrrhalta rufosanguinea* (Say) | BM704473 | PyrrufGGaCHR | Canada: Quebec, L’Outaouais, Quyon | AJ841377 | AJ841496 | AJ841625 |
|  | *Pyrrhalta viburni* (Paykull) | BM704474 | PyrvibGGaCHR | Germany: Bavaria, Oberammergau, Lain Valley | AJ841378 | AJ841497 | AJ841626 |
|  | *Sphenoraia bicolor* (Hope) | BM704475 | SphbicGGaCHR | India: Chattisgarh, Durg, Bhilai Steel Township, Sector 8 Pk. | AJ841379 | AJ841498 | AJ841627 |
|  | *Trirhabda virgata* (LeConte) | BM704476 | TrivirGGaCHR | USA: OH, Lucas Co., Maumee Bay S. P. | AJ841380 | AJ841499 | AJ841628 |
|  | Galerucinae sp. | BM704477 | XxxxxxGGaCHR | South Africa: E Cape, Tsitsikamma N.P. | AJ841381 | AJ841500 | AJ841629 |
| Cassidinae (=Cryptostoma) |  |  |  |  |  |  |  |
| Cassidini | *Agroiconota bivittata* (Say) | BM704478 | AgrbivCCaCHR | USA: OH, Pike Co., Jackson Lake | AJ841382 | AJ841501 | AJ841630 |
|  | *Aspidomorpha miliaris* (Fabricius) | BM704479 | AspmilCCaCHR | Indonesia: Sumatra, Gn Talamau, 17 km E Simpangempat | AJ841383 | AJ841502 | AJ841631 |
|  | *Cassida rubiginosa* Müller | BM704480 | CasrubCCaCHR | USA: VA, Rockingham Co., Harrisonburg | AJ841384 | AJ841503 | AJ841632 |
|  | *Charidotella sexpunctata* (Fabricius) | BM704481 | ChasexCCaCHR | Canada: Ontario, Lanark Co., Almonte | - | AJ841504 | AJ841633 |
|  | *Charidotella zona* (Fabricius) | BM704482 | ChazonCCaCHR | French Guiana: Cayenne Harbour, Mt. Rorota | AJ841385 | AJ841505 | AJ841634 |
|  | *Deloyala guttata* (Olivier) | BM704483 | DelgutCCaCHR | USA: WV, Mason Co., Bellmeade | AJ841386 | AJ841506 | AJ841635 |
|  | *Helocassis clavata* (Fabricius) | BM704484 | HelclaCCaCHR | USA: NJ, Monmouth Co., Roosevelt | AJ841387 | AJ841507 | AJ841636 |
|  | *Laccoptera nepalensis* Boheman | BM704485 | LacnepCCaCHR | China: Hong Kong Is., Jardine’s Lookout | - | AJ841508 | AJ841637 |
|  | *Laccoptera nepalensis* Boheman | BM704485bis | Lacne2CCaCHR | Indonesia: Sumatra, Gn Talamau, 17 km E Simpangempat | AJ841388 | AJ841509 | AJ841638 |
|  | *Microctenochira reticularis* (DeGeer) | BM704486 | MicretCCaCHR | French Guiana: Cayenne Harbour, Mt. Rorota | AJ841389 | AJ841510 | AJ841639 |
| Hispini | *Anisodera sp.* | BM704487 | AnixxxCHiCHR | Malaysia: Pahang, Kuala Lipis env., Kenong Pumba Pk. | AJ841390 | AJ841511 | AJ841640 |
|  | *Arescus labiatus* Perty | BM704488 | ArelabCHiCHR | French Guiana: Cayenne Harbour, Mt. Rorota | AJ841391 | AJ841512 | AJ841641 |
|  | *Baliosus nervosa* (Panzer) | BM704489 | BalnerCHiCHR | USA: NJ, Atlantic Co., Stockton College | AJ841392 | AJ841513 | AJ841642 |
|  | *Chalepus walshii* (Crotch) | BM704490 | ChawalCHiCHR | USA: WV, Greenbrier Co., Tuckahoe Lake | AJ841393 | AJ841514 | AJ841643 |
|  | *?Dactylispa sp.* | BM704491 | DacxxxCHiCHR | Indonesia: Sumatra, Gn Talamau, 17 km E Simpangempat | AJ841394 | AJ841515 | AJ841644 |
|  | *Dicladispa testacea* (Linnaeus) | BM704492 | DictesCHiCHR | Portugal: Setúbal, Murta | AJ841395 | AJ841516 | AJ841645 |
|  | *Gonophora nigrimembris* Weise | BM704493 | GonnigCHiCHR | Indonesia: Sumatra, Gn Talamau, 17 km E Simpangempat | AJ841396 | AJ841517 | AJ841646 |
|  | *Hispellinus ?coarctatus* (Chapuis) | BM704494 | HiscoaCHiCHR | Australia: QLD, Mason Creek | AJ841397 | AJ841518 | AJ841647 |
|  | *Homalispa vespertina* Baly | BM704495 | HomvesCHiCHR | French Guiana: Cayenne Harbour, Mt. Rorota | AJ841398 | AJ841519 | AJ841648 |
|  | *Imatidium capense* (Herbst) | BM704496 | ImacapCHiCHR | French Guiana: Road N2 Cayenne to Regina, Cacao (Laotian Village) | AJ841399 | AJ841520 | AJ841649 |
|  | *Microrhopala vittata* (Fabricius) | BM704497 | MicvitCHiCHR | USA: NJ, Atlantic Co., Northville | AJ841400 | AJ841521 | AJ841650 |
|  | *Octotoma scabripennis* (Guérin-Méneville) | BM704498 | OctscaCHiCHR | Australia: QLD, Brisbane, Mt. Coot-tha | AJ841401 | AJ841522 | AJ841651 |
| Spilopyrinae | *Bohumiljania caledonica* (Jolivet) | BM704499 | BohcalSpiCHR | New Caledonia: La Foa | AJ781508 | AJ781562 | AJ781625 |
|  | Hornius grandis (Philippi et Philippi) | - | HorgraSpiCHR | Chile: Valdivia | AJ781507 | AJ781561 | AJ781624 |
|  | *Spilopyra sumptuosa* Baly | BM704500 | SpisumSpiCHR | Australia: New South Wales, Murvillumbah | AJ781510 | AJ781564 | AJ781627 |
|  | *Stenomela pallida* Erichson | BM704501 | StepalSpiCHR | Chile: Concepción, Hualpén | AJ781509 | AJ781563 | AJ781626 |
| Synetinae | *Syneta adamsi* Baly | BM704502 | SynadaSynCHR | China: Hebei, Wulingshan | AJ781512 | AJ781566 | AJ781629 |
| MEGALOPODIDAE |  |  |  |  |  |  |  |
| Zeugophorinae | *Zeugophora varians* Crotch | BM704503 | ZeuvarZeuMEG | Canada: Ontario, Le Mer Bleue | AJ841402 | AJ841523 | AJ841652 |
| ORSODACNIDAE |  |  |  |  |  |  |  |
| Aulacoscelidinae | *Aulacoscelis appendiculata* Cox and Windsor | BM704504 | AulappAulORS | Nicaragua: Masaya | AJ841403 | AJ841524 | AJ841653 |
| Orsodacninae | *Orsodacne atra* (Ahrens) | BM704505 | OrsatrOrsORS | Canada: Ontario, Renfrew, Sand Point | AJ781560 | AJ781623 | AJ781686 |
| VESPERIDAE (OUTGROUP) |  |  |  |  |  |  |  |
| Vesperinae | *Vesperus sanzi* Reitter | - | VessanVesVES | Spain: Salamanca, Puebla de Azaba | - | AJ841541 | AJ841670 |
